# Correction: Evaluation of two weight stigma scales in Malaysian university students: weight self-stigma questionnaire and perceived weight stigma scale

**DOI:** 10.1007/s40519-023-01587-8

**Published:** 2023-07-22

**Authors:** Wan Ying Gan, Serene En Hui Tung, Kamolthip Ruckwongpatr, Simin Ghavifekr, Chirawat Paratthakonkun, Ira Nurmala, Yen-Ling Chang, Janet D. Latner, Ru-Yi Huang, Chung-Ying Lin

**Affiliations:** 1 Department of Nutrition, Faculty of Medicine and Health Sciences, Universiti Putra Malaysia, 43400 Serdang, Selangor Malaysia; 2 Department of Public Health Medicine, Faculty of Medicine and Health Sciences, Universiti Malaysia Sabah, 88400 Kota Kinabalu, Sabah Malaysia; 3 Institute of Allied Health Sciences, National Cheng Kung University Hospital, College of Medicine, National Cheng Kung University, 1 University Rd, Tainan, 701401 Taiwan; 4 Faculty of Education, University of Malaya (UM), 50603 Kuala Lumpur, Malaysia; 5 College of Sports Science and Technology, Mahidol University, Sala Ya, Phutthamonthon, Nakhon Pathom, 73170 Thailand; 6 Department of Health Promotion and Behavioral Sciences, Faculty of Public Health, Universitas Airlangga, Surabaya, Indonesia; 7 Department of Family Medicine, Cardinal Tien Hospital, New Taipei, Taiwan; 8 Department of Psychology, University of Hawaii at Manoa, Honolulu, HI USA; 9 Department of Family and Community Medicine, E-DA Hospital, No. 1, Yida Rd., Yanchao Dist., Kaohsiung, 82445 Taiwan; 10 School of Medicine for International Students, College of Medicine, I-Shou University, Kaohsiung, Taiwan; 11 Biostatistics Consulting Center, National Cheng Kung University Hospital, College of Medicine, National Cheng Kung University, Tainan, Taiwan; 12 Department of Occupational Therapy, College of Medicine, National Cheng Kung University, Tainan, Taiwan; 13 Department of Public Health, College of Medicine, National Cheng Kung University, Tainan, Taiwan

**Correction: Eating and Weight Disorders: Studies on Anorexia, Bulimia and Obesity (2022) 27:2595–2604** **https://doi.org/10.1007/s40519-022-01398-3**

In this article, the given and family names of the following two authors were incorrect. The correct names are given below.

- Given name is Kamolthip and family name is Ruckwongpatr.
- Given name is Chirawat and family name is Paratthakonkun.

The original article [1] has been corrected.
